# Elucidating the Interactions Between Heparin/Heparan Sulfate and SARS-CoV-2-Related Proteins—An Important Strategy for Developing Novel Therapeutics for the COVID-19 Pandemic

**DOI:** 10.3389/fmolb.2020.628551

**Published:** 2021-01-25

**Authors:** Mingjia Yu, Tianji Zhang, Wei Zhang, Qianyun Sun, Hongmei Li, Jin-ping Li

**Affiliations:** ^1^Beijing Advanced Innovation Center for Soft Matter Science and Engineering, Beijing University of Chemical Technology, Beijing, China; ^2^Division of Chemistry and Analytical Science, National Institute of Metrology, Beijing, China; ^3^Division of Chemistry, Shandong Institute of Metrology, Jinan, China; ^4^Department of Medical Biochemistry and Microbiology, University of Uppsala, Uppsala, Sweden

**Keywords:** heparin, heparan sulfate, COVID-19, coronavirus, interaction

## Abstract

Owing to the high mortality and the spread rate, the infectious disease caused by SARS-CoV-2 has become a major threat to public health and social economy, leading to over 70 million infections and 1. 6 million deaths to date. Since there are currently no effective therapeutic or widely available vaccines, it is of urgent need to look for new strategies for the treatment of SARS-CoV-2 infection diseases. Binding of a viral protein onto cell surface heparan sulfate (HS) is generally the first step in a cascade of interaction that is required for viral entry and the initiation of infection. Meanwhile, interactions of selectins and cytokines (e.g., IL-6 and TNF-α) with HS expressed on endothelial cells are crucial in controlling the recruitment of immune cells during inflammation. Thus, structurally defined heparin/HS and their mimetics might serve as potential drugs by competing with cell surface HS for the prevention of viral adhesion and modulation of inflammatory reaction. In this review, we will elaborate coronavirus invasion mechanisms and summarize the latest advances in HS–protein interactions, especially proteins relevant to the process of coronavirus infection and subsequent inflammation. Experimental and computational techniques involved will be emphasized.

## Introduction

Currently, the whole world is facing the deadly coronavirus disease 2019 (COVID-19) outbreak caused by the coronavirus (CoV) SARS-CoV-2, which has been far beyond the outbreaks caused by the other two major coronaviruses (SARS and MERS) in the past 20 years (Drosten et al., 2003; Zaki et al., 2012). So far, there are no specific therapeutic and effective drugs available. Vaccines, although achieving success worldwide, are still far from being widely accessible. In this regard, multidimensional antiviral strategies are strongly needed in preventing the spread of COVID-19 and treating infected individuals.

**Figure 1:**
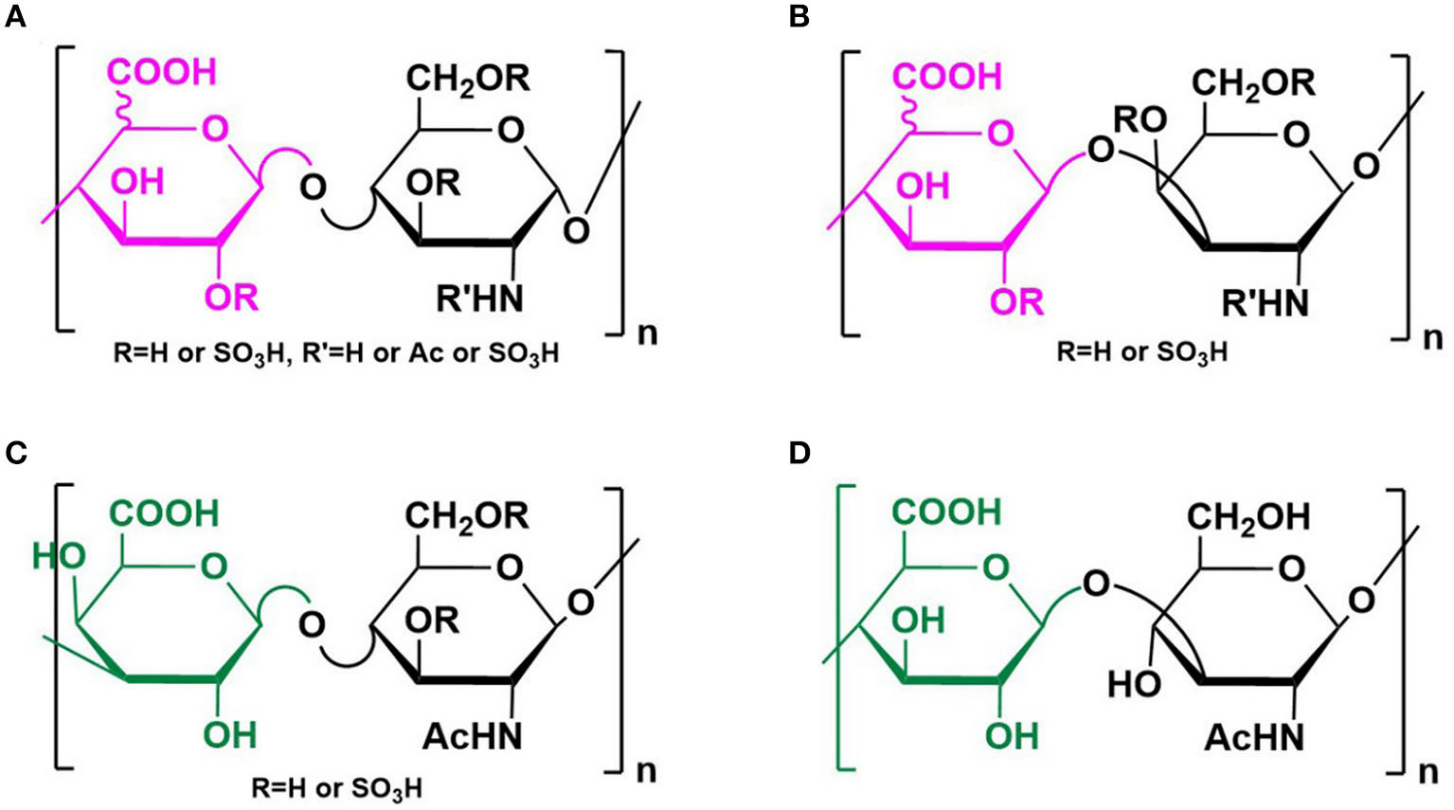
Structure of GAGs. Structures of heparin/heparan sulfate **(A)**, chondrotin/dermatan sulfate **(B)**, keratan sulfate **(C)**, and hyaluronic acid **(D)** were illustrated. Reprinted from ref Tianji Zhang et al. (2019) with permission.

Glycosaminoglycans (GAGs) are a group of anionic polysaccharides composed of repeating disaccharide building blocks, including heparin/heparan sulfate (HS) (-4GlcAβ/IdoAα1-4GlcNxα1-, x = Ac, SO_3_H or H), chondrotin/dermatan sulfate (-4GlcAβ/IdoAα1-3GalNxβ1-), keratan sulfate (-3GalAβ1-4GlcNAcβ1-), and hyaluronic acid (HA) (-4GlcAβ1-3GlcNAcβ1-) (Figure 1). Sulfation at various positions of the sugar residues could occur except for the HA, making their structures heterogeneous and extremely difficult to characterize (Tianji Zhang et al., 2019). Among them, heparin and HS exhibit the most diverse biological activities, most of which are mediated by their interactions with proteins (Li and Kusche-Gullberg, 2016). Recent work identified HS on the cell surface as a co-receptor for the SARS-CoV-2 spike protein (S protein) (Clausen et al., 2020), making the HS–S protein interaction an extremely appealing target for manipulating SARS-CoV-2 infection. Designing competing HS mimetics requires the elucidation of the mode of interaction, particularly sequence specificities of the HS.

In this review, we will summarize the classification and invasion mechanisms of the major CoVs and elaborate possible antiviral strategies based on the interactions between heparin/HS and proteins. Relevant technologies involved in elucidating heparin/HS–protein interactions are crucial for developing (sequence) specific antiviral molecules and will thus be underlined.

## Classification and Invasion Mechanisms of the Coronavirus

CoVs are highly diverse, enveloped, and positive-sense single-stranded (up to 30,000 bp) RNA viruses (Coutard et al., 2020) belonging to the *Nidovirales* order in the subfamily of *Othocoronavirinae* (Wang et al., 2020). Infection by the viruses can cause severe diseases affecting upper respiratory, gastrointestinal, and central nervous systems in humans and other animals (Gallagher and Buchmeier, 2001). Based on systematic analysis of viral nucleic acid sequence, CoVs can be classified into four genera: alpha, beta, gamma, and delta according to the 10th Report on Virus Taxonomy from the International Committee on Taxonomy of Viruses (ICTV) (Fehr and Perlman, 2015). Among them, alpha- and beta-coronaviruses can infect mammals, gamma-coronaviruses can infect avian species, while delta-coronaviruses can infect both (Li, 2016). Currently, there are seven representative strains of human coronaviruses (HCoVs) including four low-pathogenic coronaviruses [HCoV-229E, HCoV-NL63 (alpha-coronaviruses), HCoV-OC43, and HCoV-HKU1 (beta-coronaviruses)], which cause mild respiratory diseases in humans (Su et al., 2016), and three high pathogenic strains including HCoVs {severe acute respiratory syndrome coronavirus (SARS-CoV) (Drosten et al., 2003), Middle East respiratory syndrome coronavirus (MERS-CoV) (Elfiky et al., 2017), and severe acute respiratory syndrome coronavirus 2 (SARS-CoV-2) (beta-coronaviruses) (Hui et al., 2020)}. The three highly pathogenic strains have caused deadly pneumonia in humans since the beginning of the twenty-first century. Unfortunately, so far, there are still no specific therapeutics approved against these human-infecting coronaviruses, mainly due to lacking sufficient knowledge in the pathological process of viral infection. Thus, in-depth understanding of the infection mechanisms will facilitate the development of effective interventions against these highly pathogenic coronaviruses and are of high urgency for the control and treatment of COVID-19.

**Figure 2:**
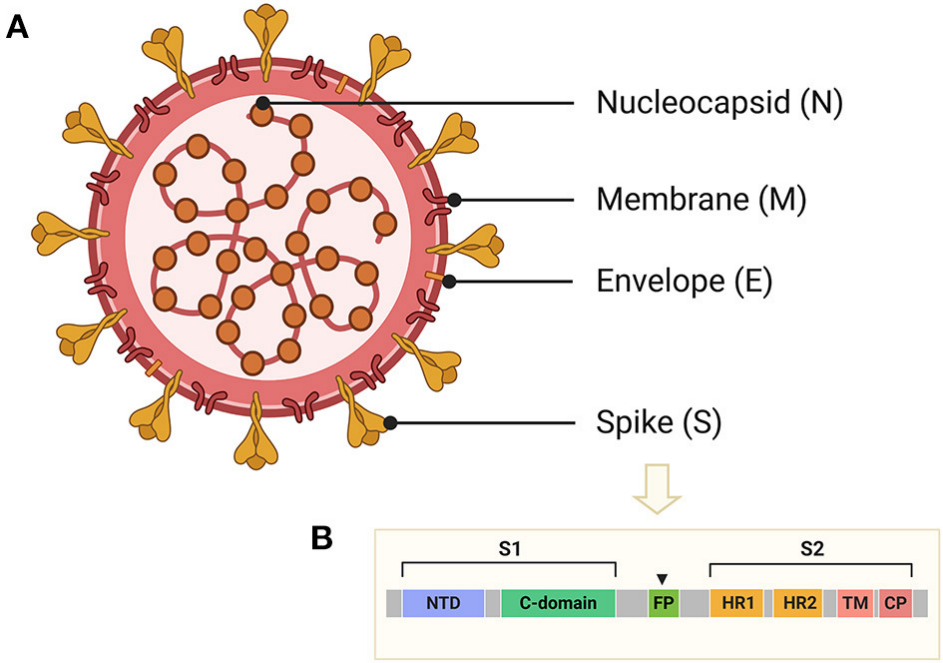
Schematic structure of CoVs and the S protein. **(A)** Schematic structure of virion of CoVs and their four structural proteins, including spike (S), envelope (E), membrane (M), and nucleocapsid (N) proteins. **(B)** Schematic representation of S protein of CoVs, including receptor-binding domain (RBD), N-terminal domain (NTD), or C-terminal domain (C-domain), fusion peptide (FP), heptad repeat 1 (HR1), heptad repeat 2 (HR2), transmembrane domain (TM), and cytoplasm domain (CP).

**Figure 3:**
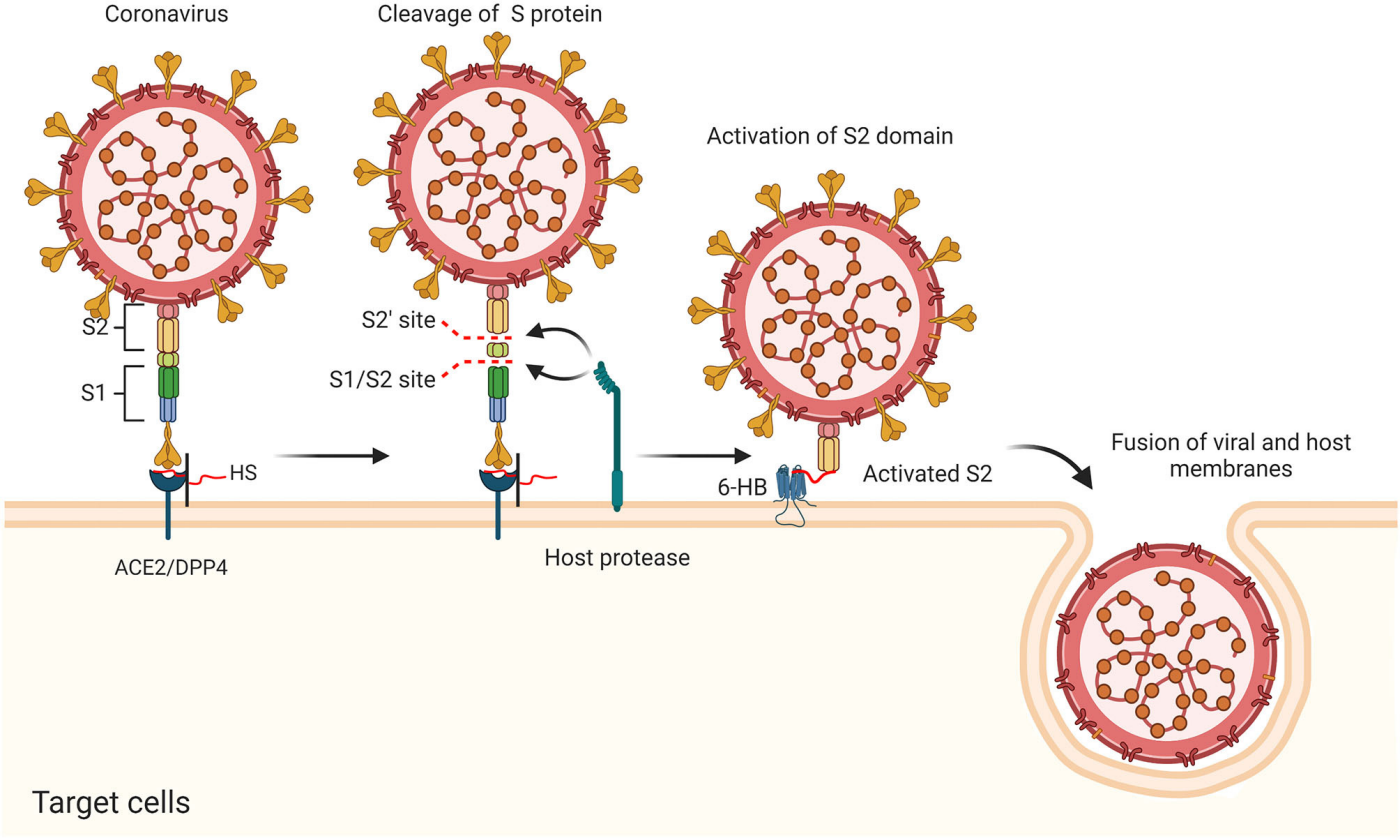
Putative antiviral mechanism of CoVs during viral entry, including the cleavage of S protein, activation of S2 domain, and the fusion of viral and host membranes.

CoVs share similar genome identities. Two-thirds of the genome at the 5′-terminus contain two large overlapping open reading frames (ORFs), ORF 1a and ORF 1b, which encode polyproteins 1a (pp1a) and pp1b/1ab, respectively. The polyproteins can be further cleaved into 15–16 non-structural proteins (nsp2-nsp16 or nsp1-nsp16). One-third of the genome at the 3′-terminus encodes four common structural proteins in the order of Spike (S) that characterizes all coronaviruses, Envelope (E), Membrane (M), and Nucleocapsid (N) (Wang et al., 2020) (Figure 2A). The S protein is a trimeric class I fusion protein that protrudes from the virion surface and mediates receptor recognition, membrane fusion, virus entry, and antibody neutralization (Gallagher and Buchmeier, 2001). Considering its significant functions during viral infection (Liu et al., 2004), the S protein serves as a main target for the development of antiviral drugs (Du et al., 2017), antibodies (He et al., 2006), entry inhibitors (Lu et al., 2014), and vaccines (Du et al., 2009). Each monomer of the trimeric S protein is ~180 kDa containing two subunits—a receptor-binding subunit (S1) and a membrane-fusion subunit (S2), which are linked through a fusion peptide. The S1 subunit contains two independent domains—the N-terminal domain (NTD) and the C-terminal domain (C-domain) (Ou et al., 2020) (Figure 2B), either of which can serve as the receptor-binding domain (RBD) depending on the virus strains (Kubo et al., 1994; Ou et al., 2017). The S2 subunit consists of four main domains—the heptad repeat 1 (HR1) domain, heptad repeat 2 (HR2) domain, transmembrane domain (TM), and cytoplasm domain (CP) (Xia et al., 2020). During viral infection, a two-step sequential protease cleavage process triggers the activation of S proteins (Belouzard et al., 2009; Millet and Whittaker, 2014), which is modulated by host range and cell tropism. The first cleavage occurs between the S1 and S2 subunits, leading to the release of the S1 subunit and its transition to the post-fusion conformation (Su et al., 2016). Then, as the RBD of the S1 subunit binds to a host cell receptor [CoVs can recognize both angiotensin-converting enzyme 2 (ACE2) and dipeptidyl peptidase 4 (DPP4, also known as CD26)] (Kuhn et al., 2004; Raj et al., 2013), another cleavage site on S2 is exposed and cleaved by host proteases at the S2' site located upstream of the fusion peptide (FP). The HR1 and HR2 domains of S2 form a six-helix bundle (6-HB) fusion core in order to bring viral and cellular membranes into close proximity for subsequent fusion and infection (Figure 3) (Bosch et al., 2004). The host proteases such as furin (Millet and Whittaker, 2014), cathepsins (Bertram et al., 2013), human airway trypsin-like protease (Berman et al., 2000; Bertram et al., 2011), and transmembrane protease serine protease-2 (TMPRSS-2) (Gierer et al., 2013) are widely expressed in many important organs, which is a critical reason for the systematic infection, serious pathogenicity, and high mortality of the CoVs. Therefore, the RBD and 6-HB fusion core of CoVs and the proteases on infected cells have become potential targets for the development of virus attachment/fusion inhibitors, neutralizing antibodies, and vaccines.

Invasion of CoVs occurs in two steps, initial binding to the receptor on the cell surface and fusion of S protein with the host cell membrane to deliver their nucleocapsid to the target cell. It has been known that CoVs often initially interact with cell surface molecules to promote their binding to specific receptors. HS proteoglycans (HSPGs) are abundantly present in almost all mammalian cells and serve as a co-receptor for a number of viruses (Gomes and Dietrich, 1982). HSPGs could initially bind to the surface proteins of CoVs, promote subsequent recognition of a secondary Receptor (ACE2/DPP4), and facilitate the attachment and entry of virus by increasing their local concentrations. Studies also suggested that different compositions in HS could impact the tropism of viruses (Wickramasinghe et al., 2011) and HS co-receptors on host cell surface leads to conformational changes of the CoVs' S proteins (Lang et al., 2011; Milewska et al., 2014; Mycroft-West et al., 2020a), possibly through the formation of a ternary complex (Clausen et al., 2020) (Figure 3). These findings suggested that the HS–S protein interactions might serve as a potential target to attenuate virus infection.

## Heparin/HS and their Interactions With Protein

### Structures of Heparin/HS

**Figure 4:**
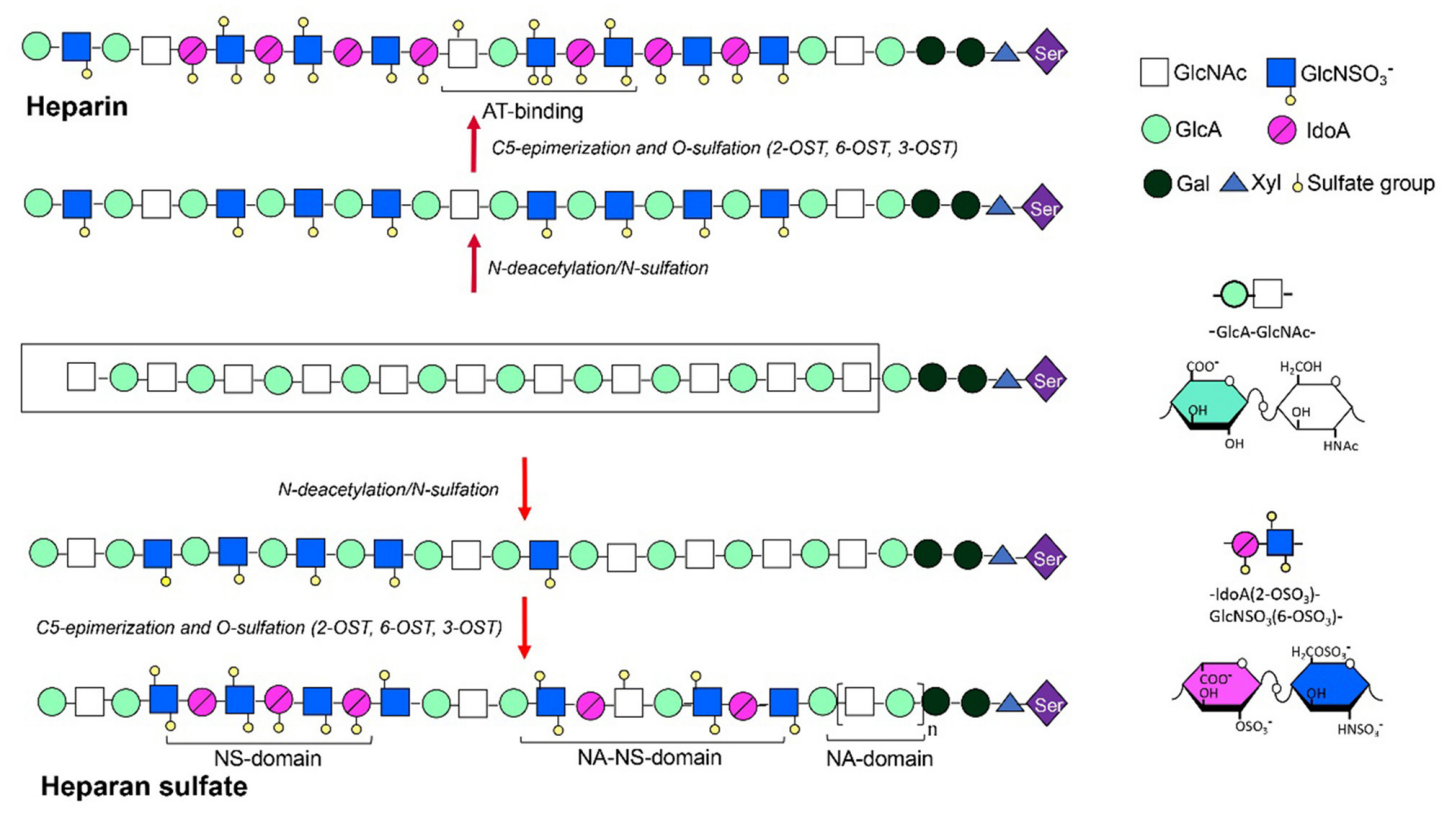
Synthesis and structures of heparin/HS.

Heparin is a significant anti-coagulant that has been used in clinic over decades. The heparin polysaccharide chains are linear and polyanionic, with repeating disaccharide units of α-L-iduronic acid (IdoA) or β-D-glucuronic acid (GlcA) residue linked to glucosamine (GlcN) residue by a 1-4 glycosidic bond. The sugar units are sulfated at N-, 6- and 3-O on the GlcN residues as well as 2-O on the hexuronic acid by site-specific sulfotransferases. The 3-O-sulfation is rare but critical for heparin to form a specific pentasaccharide domain that specifically bind to anti-thrombin with high affinity, which is essential for its anti-coagulant activity (Lindahl et al., 1980). In addition to the anti-coagulant activity, heparin and its derivatives have also been studied for their anti-inflammatory, antiviral, anti-angiogenesis, anti-neoplastic, and anti-metastatic effects (Hao et al., 2019). HS shares high structural similarity with heparin (Linhardt and Toida, 2004), but generally with lower level of sulfation and epimerization, therefore displaying distinct domain structures (Figure 4). The functionalities of heparin and HS are mediated by their interaction with various proteins including proteases, protease inhibitors, chemokines, cytokines, growth factors, and their respective receptors (Xu and Esko, 2014; Seffer et al., 2020), with variable specificities.

### An Overview of Heparin/HS and Protein Interactions

Due to the highly anionic nature of heparin/HS, the interactions between heparin/HS and proteins are primarily through the interaction between negatively charged sulfate and carboxyl groups on heparin/HS and positively charged lysine and arginine residues on the proteins. The role of electrostatics in heparin/HS–protein interactions was elucidated in several studies (Olson et al., 1991; Thompson et al., 1994; Friedrich et al., 2001). Meanwhile, nonionic interactions such as hydrogen bonding and van der Waals packing also contribute to the free energy for the binding reactions (Thompson et al., 1994).

Some heparin/HS binding proteins can be identified by amino acid sequences known as Cardin-Weintraub motifs corresponding to “XBBXBX” and “XBBBXXBX”, where X is a hydropathic residue and B is a basic residue, such as arginine and lysine, responsible for interacting with the sulfate groups present on heparin/HS (Cardin and Weintraub, 1989; Hileman et al., 1998). On the other hand, well-characterized heparin/HS–protein interactions revealed specific requirement of the carbohydrate sequence. The most prominent example is the binding of antithrombin with the unique pentasaccharide sequence, -GlcNS/Ac6S-GlcA-GlcNS3S6S-IdoA2S-GlcNS6S- in heparin, where the 3-O-sulfation is critical (Richard et al., 2009). Unlike the extremely rigorous sequence requirement as of antithrombin, or the purely non-specific interaction as in the case of heparin and protamine (Hubbard and Jennings, 1985), majority of the heparin/HS–protein interactions are selective. For instance, the interaction between HS and FGF2, a member of the fibroblast growth factor family, prefers the disaccharide unit of IdoA2S and GlcNS on heparin/HS (Turnbull et al., 1992; Jemth et al., 2002). More evidence is emerging, indicating that binding of HS and proteins is somewhat between purely specific and generally non-specific (Forsten-Williams et al., 2008; Nugent et al., 2013). Non-specific bindings solely depend on the high negative charge density of the carbohydrate chain and positively charged residues of the proteins, while modifications or domains on heparin/HS determine specificity levels of the interactions (Xu and Esko, 2014).

### Experimental Technologies Involved in Studying Heparin/HS–Protein Interactions

The major challenge for elucidating heparin/HS–protein interactions is to decipher the carbohydrate sequence that is commonly of high heterogeneity. Multidimensional technologies that facilitate understanding sequence specificities have been comprehensively summarized in a recent review (Yang and Chi, 2017), including X-ray crystallography, nuclear magnetic resonance (NMR) spectroscopy, and mass spectrometry (MS).

Heparin/HS oligosaccharide microarrays are valuable tools that can be used to probe the interactions between structurally defined oligosaccharides and proteins with relatively small amounts of samples. The bottleneck of the microarray assay is to synthesize oligosaccharide libraries of intensive diversity. Zong et al. prepared a tetrasaccharide library consisting of 47 unique structures, which is one the most comprehensive HS microarrays covering a large portion of possible structural variabilities (Zong et al., 2017). In a recent study, chemoenzymatic strategies have been successfully applied to construct microarrays composed of tetrasaccharide to 18-mer containing various N-, 6-O-, 2-O-, and 3-O-sulfation modifications (Horton et al., 2020). Cell-based microarrays have also been developed, aiming at demonstrating the functionality of specific heparin/HS saccharides in real cell signaling (Puvirajesinghe et al., 2012; Sterner et al., 2013).

The surface plasmon resonance (SPR) sensor (Thompson et al., 1994) is one of the most convenient tools for detecting heparin/HS–protein interactions through changes of the refractive index signals. One of the major advantages of SPR is the capacity of probing biomolecular interactions at the thermodynamic level, offering real-time and label-free measurement of reaction rate constants (*k*_on_, *k*_off_) and resultant equilibrium constants (*K*_A_, *K*_D_) (Homola, 2008). In a recent study, interactions between heparin/HS and various cytokines were characterized by coupling surface plasmon resonance imaging for thermodynamic analysis method and Matrix-Assisted Laser Desorption/Ionization Time of Flight Mass Spectrometry (MALDI-TOF-MS) for structural determination (Przybylski et al., 2020). A self-assembled monolayer of short polyethylene oxide chains was used for grafting cytokines. Captured carbohydrates were carried out directly on the biochip surface using MALDI-TOF-MS, while MS identification was enhanced by on-chip digestion of the cytokine-bound GAGs by heparinase treatment.

### Computational Techniques

Despite the advances in the experimental techniques, there are limitations (e.g., failure in the acquisition of co-crystal structures) in obtaining the information regarding molecular interactions. Thus, computational techniques are indispensable tools for comprehensively understanding the heparin/HS–protein interactions. On the basis of theoretical models, the computational techniques are especially helpful in designing novel drug, performing wide scale analysis against large database (e.g., the PDB database), and for understanding the interaction dynamics. The interactions between protein and heparin/HS can be weak or strong, transient or stable, non-permanent or permanent, which can be evaluated by basic parameters of binding affinities such as the equilibrium dissociation constant, *K*_D_ (Ma et al., 2018); Gibbs free energy of binding, Δ*G* (Steinbrecher and Labahn, 2010); inhibition constant, K_i_ (Pekkarinen et al., 2007); half maximal inhibitory concentration, IC_50_ (Sebaugh, 2011); and electrostatic potential energy (Bitencourt-Ferreira et al., 2019). Several techniques are available for a wide application in assessment of protein-heparin/HS interaction.

#### Homology Modeling

The homology modeling method uses 3D structures deposited in the PDB database to predict protein structures of sequential similarities. Homology modeling can give spatial structures with the highest accuracy (Werner et al., 2012) and thus has been widely applied for rational analysis of interactions between small organic molecule (ligand) and target protein during the docking and virtual screening for drug discovery (Cheng et al., 2012). Homology modeling can be built by four methods, including rigid body assembly [by tools like SWISS-MODEL (Arnold et al., 2006)], segment matching [by tools like SEGMOD/ENCAD (Levitt, 1992)], spatial restraint [by tools like MODELER (Sali and Blundell, 1993)], and artificial evolution [by tools like NEST (Petrey et al., 2003)].

#### Molecular Docking

Molecular docking is a computational procedure extensively used in novel drug discovery, through which a small molecule (ligand) is docked into a macromolecule (target protein) at the binding sites to predict the binding conformations and affinity. The conformation of a ligand binding with the receptor depends on its state variables (including position: *x*-, *y*-, and *z*-translations; orientation: euler angles, axis angle, and quaternion; and conformation: torsion angles for each rotatable bond), which decides the extent of the multidimensional search space within the protein–ligand interaction. All docking methods require a scoring function to rank various candidate protein–ligand binding modes and a search method to explore their state variables. Scoring functions are computational approximations to predict protein–ligand binding affinity based on empiricism, force field, and knowledge, while search methods are classified into local and global ones by the extent of search space. Local search methods [such as solis and wets (Solis and Wets, 1981) and the pattern search (Lewis and Torczon, 2002)] tend to find the nearest or local minimum energy to the current conformation, whereas global methods [such as Monte Carlo (MC) simulated annealing (SA) (Kirkpatrick et al., 1983) and the genetic algorithm (GA) (Goldberg, 1988)] search for the best or global minimum energy within the defined search space. Hybrid global-local search methods have been shown to perform even better due to the higher efficiency in finding lower energies among the different candidate protein–ligand binding modes (Morris and Lim-Wilby, 1999). Molecular docking can be performed by various docking programs such as AutoDock (Goodsell et al., 1996), AutoDock Vina (Trott and Olson, 2010), FlexX (Rarey et al., 1996), GOLD (Jones et al., 1997), and Molegro Virtual Docker (MVD) (De Azevedo, 2010).

#### Electrostatic Potential Energy

Since electrostatic interactions are part of scoring functions that widely influence the binding affinity of protein–ligand complexes, electrostatic potential energies are calculated in computational models to compare protein–ligand binding affinities. The electrostatic force is conservative as it only depends on the initial and final positions and most protein–ligand complexes show only partial charges. Partial Equalization of Orbital Electronegativity (PEOE) is the most widely used method (Gasteiger and Marsili, 1980) provided by the software AutoDockTools4 (Morris et al., 2009) that estimates partial charges of target protein and ligands in order to calculate electrostatic potential energy.

#### Molecular Dynamics

Molecular dynamics (MD) is a computational simulation technique that can obtain not only multiple conformations of target proteins and ligands, but also a wealth of energetic status about the interactions in a time-dependent manner. MD simulations combined with binding free energy calculations can improve the accuracy of binding prediction and are thus suitable for studying the motions of target protein on ligand binding (Radkiewicz and Brooks, 2000; Salsbury et al., 2001). The Newtonian equation of motions is applied for each atom in the MD simulations for approximations (Schlick, 2010), which requires the information of initial coordinates (obtained from experimental structures, models, or combination of the two), potential (obtained from different force fields along with the coordinates) (MacKerell et al., 1998), and algorithms. Given the diverse complexity of the protein–ligand structures, different force field models [such as CHARMM (Miller et al., 2008), AMBER (Guvench and MacKerell, 2008), and GROMACS (Van Der Spoel et al., 2005)] are flexibly used during simulation, which are associated with modeling suites of CHARMM (Brooks et al., 2009), AMBER (Case et al., 2005), GROMACS (Hess et al., 2008), and NAMD (Phillips et al., 2005). Owing to the advances in computers and algorithms, the complexes of biomacromolecules can be simulated in nanoseconds with whole atoms, generating numerous conformations. The characterization of each conformation is accomplished by sophisticated methods that could be divided up to four types, including gross measures of protein and simulation stability, clustering analysis, quasiharmonic and principal component analysis, and correlation function analysis. The gross measures of protein and simulation stability is the most widely used approach for checking simulation integrity and estimating equilibration timescale of the simulation. Parameters such as root-mean-square deviation (RMSD), structural clustering, free energy of binding and native contacts, and average temperature and pressure are generally calculated for obtaining their fluctuations.

Docking an HS fragment of proper size (≥4 monosaccharide units) to a protein is challenging due to the flexibility brought by the glycosidic rings, linkages, and the high density of negative charges. Sapay et al. proposed a two-step method based on molecular docking and MD simulation to explore the binding modes of HS to cellular growth factors (FGF2 and CXCL12α) (Sapay et al., 2011). The method provided dynamical modeling of the protein–ligand complex by building the docking models of HS fragment on protein surface and refining the contacts between HS fragment and the protein.

#### Computational Study of SARS-CoV-2 Infection

The powerful tools of computational technology has made significant contribution to the studies on viruses including SARS-CoV-2. The method of homology modeling has made initially important contribution. Based on the rich genomic information and bioinformatics analysis of the proteins encoded by the novel coronavirus genes, Wu *et al*. built 19 structures of SARS-CoV-2 by homology modeling through the Fold and Function Assignment System server, including viral papain like protease (PLpro), main protease (3CLpro, also named 3-chymotrypsin-like protease), RNA-dependent RNA polymerase (RdRp), helicase, and S protein (Wu et al., 2020). As the most efficient way to find anti-SARS-CoV-2 drugs is to screen those that are commonly used in clinic, small-molecule compounds from several resources including the U.S Food and Drug Administration (FDA)-approved drug database (ZINC drug database, ZDD), traditional Chinese medicine/natural products database, and the antiviral drugs database were docked into these computational models by ICM 3.7.3 modeling software (MolSoft LLC) to virtually screen potential druggable targets. Successfully predicted targets and potential drug compounds can be further tested *in vitro* and *in vivo* for treating SARS-CoV-2 infections.

The calculations of electrostatic potential energy were performed to estimate protein-heparin/HS binding affinities combined with the docking technique. Clausen et al. calculated the electrostatic potential map of both SARS-CoV-1 RBD (PDB ID: 3BGF) (Pak et al., 2009) and SARS-CoV-2 RBD (PDB ID: 6M17) (Yan et al., 2020) by the Molecular Operating Environment (MOE) software (Clausen et al., 2020). Combined with docking studies of oligosaccharide fragments derived from heparin with RBD, it revealed an extended electropositive surface of RBD composed of positively charged residues including R346, R355, K444, R466, and possibly R509 that could coordinate the electronegative oligosaccharides through hydrogen bonds and hydrophobic interactions. This study demonstrated that the SARS-CoV-2 S protein may mediate an enhanced interaction with HS analogs and heparinoid derivatives compared to SARS-CoV-1 by the evolution of Lys444 and Glu354 (SARS-CoV-1) to Thr444 and Asn354 (SARS-CoV-2), respectively.

MD simulations have also been proven as a convenient method to describe the motions and binding affinities of ligand into target proteins. Han et al. designed and simulated several potential peptide inhibitors (including virus-binding domain α-helices extracted from the protease domain of ACE2) against the SARS-CoV-2 coronavirus (Han and Kral, 2020). Classical MD simulations were performed by the modeling suites NAMD (Phillips et al., 2005) and CHARMM36 protein force field (MacKerell et al., 1998), which screened the most suitable peptide inhibitor with good binding affinity yet low RMSD for critical amino acids, indicative of relatively high binding energies. The novel designed peptide inhibitors have provided insights for researchers to develop therapeutic antiviral inhibitors by offering the α1 helix of ACE2 a sulfated ligand. Other molecules of similar structures, the heparin/HS for instance, could also attach to positively charged residues at the bottom of the RBD.

The timescale of the MD simulations is also a determinant for the convergence of structural clustering, free energy of binding, and native contacts between the GAGs and target proteins. Bojarski et al. analyzed the structure of fibroblast growth factor 1 (FGF1) complexed with heparin [PDB ID: 2AXM (DiGabriele et al., 1998)] through microsecond-scale simulations by the force field of AMBER16 (Bojarski et al., 2019). The analysis revealed a conformational selection mechanism of GAGs binding and determined the structural specificity in the FGF1–heparin complex. Their findings could potentially contribute to the development of novel biomaterial resembling GAGs in the field of regenerative medicine.

## Utilizing Heparin/HS–Protein Interactions to Explore Novel Strategies for Treatment of SARS-CoV-2 Infections

### Interactions Between Heparin/HS and the S Protein

Based on the knowledge of virus–heparin/HS interaction, it is assumed that exogenous added heparin/HS may interfere with viral infection. Several excellent studies have focused on the interactions between heparin/HS and the SARS-CoV-2 S protein, especially structure specificity of the carbohydrate chains. Nevertheless, it is worth noting that results from different research groups exhibited inconsistency to some extent, even if the similar analytical methods were conducted. This could be possibly attributed to experimental parameter setting, and the complexity and heterogeneity of the heparin/HS structures.

Using the SPR technique, Mycroft-West et al. first reported the SARS-CoV-2 S1 RBD binding to unfractionated heparin (Mycroft-West et al., 2020a). Through circular dichroism (Martino et al., 2020) spectroscopy, the authors further indicated that the RBD underwent conformational change in the presence of heparin, including helix and beta-sheet content alterations. The changes demonstrated that the RBD interacted with heparin in aqueous solution of physiological significance, whereby the major changes induced by heparin were those associated with antiparallel and helix content. In a subsequent study (Mycroft-West et al., 2020b), the authors found that the addition of heparin to Vero cells between 6.25 and 200 μg ml^−1^ inhibited invasion of SARS-CoV-2 by 44–80%. Additionally, SPR data revealed that 2-O, 6-O, and completely desulfated heparin had no inhibitory activity on heparin–RBD binding, proving the significance of 2-O and 6-O-sulfation on heparin/HS-spike interactions. On the other hand, persulfated, N-desulfated/N-re-acetylated, and strikingly 2-O/6-O doubly desulfated heparin possessed inhibitory activity. The authors attributed this phenomenon to a preference of RBD for a particular spatial arrangement of charged groups.

In another study, Kim et al. found that both monomeric and trimeric SARS-CoV-2 S proteins bound to immobilized heparin (*K*_D_ = 40 and 73 pM, respectively) more tightly than the SARS-CoV and MERS-CoV S proteins (500 and 1 nM, respectively) (Kim et al., 2020). Heparin-derived oligosaccharides (dp 4 to 18), N-desulfated, 2-O-desulfated, and 6-O-desulfated heparin failed to compete with immobilized heparin for binding to the S protein, suggesting that chain length and all the sulfate groups within heparin were critical in the interaction. On the other hand, in responses to heparin, tri-sulfated non-anti-coagulant HS, and non-anti-coagulant low-molecular-weight heparin (LMWH), the binding of SARS-CoV-2 S protein to the surface-immobilized heparin decreased in a concentration-dependent fashion. The IC_50_ were determined to be 0.056, 0.12, and 26.4 μM, respectively. Additionally, unbiased computational ligand docking predicted putative heparin/HS-binding motifs on the S protein: 453–459 (YRLFRKS), 681–686 (PRRARS), and 810–816 (SKPSKRS), among which the 681–686 (PRRARS) site between the S1 and S2 subunits was a novel insertion not present in the SARS and MERS S proteins.

Liu et al. performed microarray binding experiments using an extensive HS oligosaccharide library (Liu et al., 2020). Their data suggested that the SARS-CoV-2 S protein can bind HS in a length- and sequence-dependent manner, while hexa- and octasaccharides composed of IdoA2S-GlcNS6S repeating units were identified as optimal ligands. Notably, 3-O-sulfation on the GlcN residue was proven not essential for efficient binding. In support of the microarray data, SPR experiments showed that the SARS-CoV-2 S protein bound with higher affinity to heparin (*K*_D_ = 55 nM) compared to the RBD (*K*_D_ = 1 μM) alone. The previously determined octasaccharide composed of IdoA2S-GlcNS6S repeating subunits could inhibit heparin–S protein interaction with an IC_50_ of 38 nM. Their data supported a model in which the RBD of the SARS-CoV-2 S protein conferred sequence specificity and agreed with Kim et al. (2020) where an additional HS binding site in the S1/S2 proteolytic cleavage site could enhance the avidity of binding.

To obtain insights into heparin/HS–S protein binding and virus infection in a safer circumstance, Tandon et al. pseudotyped SARS-CoV-2 S protein on a third-generation lentiviral (pLV) vector for testing the impact of various sulfated polysaccharides on transduction efficiency in mammalian cells (Tandon et al., 2020). The pLV vector pseudotyped the S protein efficiently and produced high titers on HEK293T cells. Both unfractionated heparin (UFH) and enoxaparin (a low-molecular-weight heparin drug) exhibited high apparent inhibitory activity. However, in contrast with previous SPR results, the authors found that selective desulfation at the 6-O-position of the GlcN residue did not significantly reduce inhibitory activity of either UFH or enoxaparin. Concentration–response curves showed that pLV-S particles were efficiently neutralized by a range of concentrations of UFH, enoxaparin, 6-O-desulfated UFH, and 6-O-desulfated enoxaparin with an IC_50_ of 5.99 μg/L, 1.08 mg/L, 1.77 μg/L, and 5.86 mg/L, respectively. This study also enabled SPR analysis using pseudotyped lentiviral virions instead of isolated S protein, which was of more biological relevance. In the binding competition experiments, soluble heparin, non-anti-coagulant heparin, and a non-anti-coagulant low-molecular-weight heparin (NACH) showed IC_50_ values of 125 nM, 500 nM, and 25 μM, respectively.

Tiwari et al. used a model of cellular cell-to-cell fusion assay to show that the SARS-CoV-2 S protein-mediated cell-to-cell fusion could arise even in the absence of ACE2 (Tiwari et al., 2020). Further, they demonstrated that the S protein differentially recognized the 3-O-sulfated HS structures generated by the two different isoforms, 3OST-3B and 3OST-5, and that the S2 subunit was critical for cell-to-cell fusion mediated by the S protein−3-O-sulfated HSPG pathway. SPGG, a synthetic, small, and highly sulfated non-sugar compound, was capable of serving as an effective inhibitor of cell-to-cell fusion.

In an elegant study, Clausen et al. demonstrated the dependence of HS on SARS-CoV-2 infection (Clausen et al., 2020). Molecular modeling identified the putative binding surface for oligosaccharides that resided in the RBD of the S protein and were adjacent to, but separate from the ACE2 binding site. Interactions between RBD/ectodomain and HS were proved by affinity-based approaches. A ternary complex of heparin, ACE2, and the S protein was demonstrated by binding of S protein to immobilized heparin-BSA and titrating with biotinylated ACE2, in which case the binding of ACE2 increased in proportion to the amount of S protein bound to the heparin-BSA. Through flow cytometry, the authors proved that HS was essential for the spike ectodomains binding to ACE2 and several human cell lines, while heparin lyases treatment dramatically reduced binding. Similarly, targeting heparin/HS synthesis enzymes including NDST1, HS6ST1, HS6ST2, and B4GALT7 (required for GAG assembly) significantly reduced binding. Consistent with Liu et al. (2020), the authors claimed that the interaction between heparin and the S protein was independent of the anti-coagulant activity. Furthermore, infection of pseudotyped vesicular stomatitis virus (VSV) expressing the full-length S protein and SARS-CoV-2 virus was proven to be dependent on cellular HS. Interestingly, Hep3B cells with inactivated HS6ST1/2 responded differently to VSV and SARS-CoV-2.

Relevant to the previously established dependency of HS on SARS-CoV-2 infection, Martino et al. showed that commensal host bacterial communities capable of modifying HS changed with host age and sex in adult COVID-19 patients. The prevalence of those bacteria and the expression of key microbial glycosidases, which were capable of blocking SARS-CoV-2 S protein binding to human lung adenocarcinoma cells *in vitro*, was lower in bronchoalveolar lavage fluid (BALF) compared to healthy controls (Martino et al., 2020). Zhang et al. performed a drug repurposing screen and identified Mitoxantrone, an FDA-approved cancer treatment drug that also directly targets HSPGs and inhibits pseudo-coronavirus infection (Zhang et al., 2020a). Several other drugs, Sunitinib BNTX and Latrunculin, which disrupt actin dynamics on the cell surface, were also proven to inhibit SARS-CoV-2 cell entry. The fact that structurally unrelated actin inhibitors all blocked coronavirus entry strongly suggested that the endocytosis of coronavirus required the actin cytoskeleton in addition to ACE2 and HS.

### Interactions Between Heparin/HS and Cytokines

Severity of SARS, MERS, and COVID-2019 are associated with the presence of lymphopenia and inflammatory cytokine storm (de Wit et al., 2016; Tan et al., 2020; Zhou et al., 2020). The process of inflammation storm is divided into three steps accompanied by a series of inflammatory responses and recruitment of leukocytes on the infected areas. (1) After initial invasion of virus, macrophages and mast cells immediately release macrophage inflammatory protein 1-α (TNF-α) and interleukin-1 (IL-1) at the site of pathogen adhesiveness in order to activate leukocyte extravasation. (2) Selectins (E-, L-, and P-selectins) on leukocytes interact with endothelial surface-associated HS, which allows leukocyte tethering and rolling along vessel wall (Wang et al., 2002). (3) An array of inflammatory chemokines and cytokines are activated by HS presented on the endothelial surface, which triggers integrin adhesion molecules binding onto leukocytes and subsequent leukocyte extravasation out of the blood vessel (Butcher, 1991; Norgard-Sumnicht and Varki, 1995; Tanaka et al., 1996; Luo et al., 2001). Rich evidence has shown that various inflammatory chemokines and cytokines including microphage inflammatory protein (MIP-1α), RANTES, granulocyte colony-stimulating factor, interferon-γ-inducible protein 10, monocyte chemoattractant protein 1, tumor necrosis factor-α (Kuschert et al., 1999; Huang et al., 2020), as well as IL-2 (Najjam et al., 1998), IL-7 (Borghesi et al., 1999), IL-8 (Spillmann et al., 1998), and IL-10 (Salek-Ardakani et al., 2000) selectively bind to distinct domains of the heterogeneous HS with various affinities and sequence specificities. Gao et al. reported that periodate-oxidized, borohydride-reduced heparin (RO-heparin) could inhibit thioglycollate-induced peritoneal inflammation by preventing neutrophil recruitment dependent on the release of L- and P-selectin (Gao et al., 2005). This is an indication that RO-heparin could attenuate L- and P-selectin-mediated acute inflammation.

### Current/Potential Clinical Applications of Heparin/HS in COVID-19

With the evidence described above, it is most likely that disrupting HS–protein interactions by exogenous and competitive heparin/HS mimetics could interfere with virus infection and/or suppress the inflammatory responses. In fact, COVID-19 patients commonly suffer from hyper-coagulopathy and are routinely treated with heparin/LMWH. Significant differences in 28-days mortality were observed in the subgroup of patients with a concentration of D-dimer (>3 μg/ml) higher than sixfold of the normal upper limit, or who had a sepsis-induced coagulopathy (SIC) score ≥4 (40.0 vs 64.2%, *P* = 0.029) (Shi et al., 2020a). Recent studies have shown that hospitalized patients with severe COVID-19 treated with LMWH or fondaparinux (an ultra-low-molecular-weight heparin) had better prognosis in relation to mortality (Lin et al., 2020; Russo et al., 2020; Tang et al., 2020). It needs to be noted that side effects like heparin-induced thrombocytopenia (HIT) have also been reported in heparin-treated COVID-19 patients (Daviet et al., 2020; Lozano and Franco, 2020). Correct dosage and real-time monitoring of the anti-Xa activity are crucial in heparin/LMWH treatment of COVID-2019 (Duranteau et al., 2018).

Apart from its anti-coagulant effects, a retrospective cohort study found that IL-6 levels were significantly reduced while the percentage of lymphocytes was remarkably increased in the hospitalized COVID-19 treated with LMWH in comparison to the non-LMWH-treated group (Shi et al., 2020b), demonstrating the anti-inflammatory activity of the drug. In addition to the systemic administration, local application of heparin/LMWH through intranasal or inhalation route have also been reported for the treatment of lung diseases and inhalation injury (Yildiz-Pekoz and Ozsoy, 2017; Zielinski et al., 2019). Considering the antiviral activities of heparin/LMWH, along with data suggesting that the nasal epithelium is a portal for initial infection and transmission, Tandon et al. suggested that intranasal administration of UFH may be an effective and safe prophylactic treatment SARS-CoV-2 transmission. Due to the low bioavailability of intranasally administered heparin (Bendstrup et al., 2002), this approach might avoid dangerous side effects or complications with anti-coagulation treatments while potentially still providing a prophylactic or therapeutic benefit (Tandon et al., 2020).

## Conclusive Remarks

Despite the well-established anti-coagulant activity and the observed anti-inflammatory effects, the potential anti-SARS-CoV-2 activity of heparin/HS was only recently proposed. It is still controversial regarding the structure specificities of the heparin/HS chains for its interaction with the S protein; however, *in vitro* experiments and some clinical data have provided promising evidence of heparin/HS (including their mimetics) and heparin/HS-interacting molecules as anti-SARS-CoV-2 drugs. Further elucidation of the heparin/HS–S protein interaction will facilitate the construction of structurally defined oligosaccharide sequences that can be prepared through several methods reported (Roy et al., 2014; Hansen et al., 2015; Baytas and Linhardt, 2020; Zhang et al., 2020b). Non-anti-coagulant heparin, the anti-coagulant activity of which is selectively eliminated, may also be an option to be explored for the treatment of COVID-19 patients without the risk of bleeding complications (Cassinelli et al., 2020; Lindahl and Li, 2020)

## Author Contributions

MY and TZ contributed equally to this work by writing the first draft. J-pL contributed to editing the manuscript. All authors contributed to the article and approved the submitted version.

## Conflict of Interest

The authors declare that the research was conducted in the absence of any commercial or financial relationships that could be construed as a potential conflict of interest. The handling editor declared a past collaboration with several of the authors TZ, HL, and J-pL.
